# Study on the Enhancement of Immune Function of Astaxanthin from *Haematococcus pluvialis*

**DOI:** 10.3390/foods10081847

**Published:** 2021-08-10

**Authors:** Qingsheng Fan, Zhan Chen, Yating Wu, Jiangxin Zhu, Zhou Yu

**Affiliations:** Sino German Joint Research Institute, Nanchang University, Nanchang 330006, China; cnfoods@163.com (Q.F.); cz18870029458@163.com (Z.C.); ting88484@163.com (Y.W.); 18435997128@163.com (J.Z.)

**Keywords:** *Haematococcus pluvialis*, astaxanthin, cellular immunity, humoral immunity

## Abstract

This study was aimed at investigating the effect of astaxanthin on the immune function and its safety in mice. It was administered once daily at low, medium and high doses (4.2, 8.35, 16.70 mg/kg BW) to mice for 30 days. Subsequently, the spleen and thymus index, spleen lymphocyte transformation activity, delayed allergy reaction, amounts of antibody-producing cells, half-hemolytic value HC_50_, carbon particle clearance rate, macrophage phagocytosis, and natural killer cell (NK) activity were determined. Acute oral toxicity and genotoxicity tests were conducted to evaluate the safety of astaxanthin. Compared with the control group, medium and high doses of astaxanthin significantly increased the proliferation and transformation activities of spleen lymphocytes, activities of antibody-producing cells, serum hemolysin levels, and carbon particle clearance rate in mice (phagocytic index). High doses significantly improved delayed allergy reaction and NK cell activity. Results of acute oral toxicity and genotoxicity tests were negative. Gross anatomical observations and histopathological examination showed no abnormal changes associated with the treatments. In the article, it is confirmed that astaxanthin treatments significantly improve immune functions and show no toxic effects in the experimental doses.

## 1. Introduction

Astaxanthin is a dark-pinkish non-provitamin A carotenoid. It has a chemical structure similar to that of β-carotene and can be either chemically synthesized or naturally isolated [1,2]. Synthetic astaxanthin has a low price and purity and is mainly used for feed preparations and animal feeding [3,4]. Two methods, direct and indirect, are developed for synthesizing astaxanthin. The direct method uses common carotenoid monomers for synthesis. The indirect method is through oxidation of carotenoid precursors. Regardless of the method chosen, most of the synthesized products are in *cis* configurations (natural astaxanthin is in the *trans* structure [5]). At present, only Roche in Switzerland and BASF in Germany use chemical synthesis to produce astaxanthin with a yield of only 5.0%. Piscine Energetics synthesizes astaxanthin from α-ionone. The astaxanthin isolated from natural sources has high price and purity, which is suitable for human use. There are three main sources of natural astaxanthin. First, exoskeletons of shrimps and crabs are used to extract astaxanthin after fermentation by lactic acid bacteria. However, the composition of collected shrimp exoskeletons is complicated, and the content of astaxanthin is relatively low. It is difficult to extract astaxanthin at a large scale, and other harmful substances may be included in the collected materials, which may affect the quality of astaxanthin. Second, *Phaffia rhodozyma* yeast can also produce astaxanthin, and the average content of astaxanthin in the natural red yeast is 0.40%. The yield from the red yeast is low, and the fermentation process is complicated, which leads to special requirements for equipment sets [6]. These shortcomings limit the application of this production method [7]. Currently, the method drawing the most attention and being investigated is the extraction of astaxanthin from *Haematococcus pluvialis* [8].

*Haematococcus pluvialis* is a species of monocellular freshwater green algae widely distributed in nature. It belongs to *Haematococcaceae* in *Volvocales.* The amounts of accumulated carotenoids can reach 2–5% of the dry weight of cells. It has the highest content of astaxanthin currently found and is regarded as the “concentrated product” of natural astaxanthin [9,10]. Therefore, *Haematococcus pluvialis* has become a hotspot for researchers who would like to improve the yield of astaxanthin extraction and its enrichment efficiency [11].

Astaxanthin has a variety of physiological functions. The most studied one is its powerful antioxidant effect. It is considered to be the strongest natural product with antioxidant activity. Its antioxidant potency is 100 times higher than that of vitamin E (V_E_), 10 times that of carotenoids. Astaxanthin is a kind of a conjugated double-bond chain compound with unsaturated ketone and hydroxyl groups. The carbon skeleton in the structure consists of the central polyene chain and the aromatic rings on both sides. Each aromatic ring has one hydroxyl group (OH) and one ketone group (=O), a chemical structure that can attract more unpaired electrons [12]. It has been thought that the high antioxidant activity of astaxanthin is probably due to the activation of hydroxyl groups by ketone groups, which promotes the transfer of hydrogen to peroxide radicals [13]. In addition to its antioxidant effects, astaxanthin has other physiological effects. It can reduce the level of TNF-ɑ by inhibiting the NF-KBp65 or SHP-1 signaling pathway, resulting in anti-inflammatory effects [14,15]. It has been shown that it can inhibit lipid peroxidation and DNA damage, exerting neuroprotective effects. In addition, astaxanthin has been shown to affect the processes of tumor cell production, growth and apoptosis [16,17].

Due to the unique chemical structure of astaxanthin, astaxanthin research has been mainly focused on its antioxidation effect and the underlying mechanisms. The research on the impact of the immune function has been limited. This research was set and performed to investigate the roles of astaxanthin in enhancing immune functions.

## 2. Materials and Methods

### 2.1. Samples and Animals

Astaxanthin is derived from *Haematococcus pluvialis* with a content of 1.5%, which is directly soluble in corn oil.

SPF Kunming female mice, 18–22 g, were obtained from the Laboratory Animal Center of Guangxi Medical University, laboratory animal production license No. SCXK (Gui) 2014-0002, laboratory animal quality certificate No. 45000300000475, animal laboratory (barrier facility) license No. SYXK (Gui) 2011-0005. The temperature and humidity of the animal feeding room were 20–25 °C and 55–70%, respectively.

Other than the acute toxicity test, the Ames test, the micronucleus test of bone marrow cells and the sperm abnormality test in mice were performed; the experimental groups were as described below. Two hundred healthy female Kunming mice of the SPF grade weighing 18–22 g had been bred by the Laboratory Animal Center of Guangxi Medical University and divided into five groups. Each group contained 40 mice. Immune group I was used for the ConA-induced splenic lymphocyte transformation and NK cell activity tests. Immune group II was used for the delayed allergy test. Immune group III was used for the viscera/body ratio, serum hemolysin and antibody-producing cell count tests. Immune groups IV and V were used for the carbon particle clearance test and the peritoneal macrophage-mediated phagocytosis of chicken red blood cells test, respectively.

### 2.2. Acute Toxicity Test

To conduct the maximum tolerated dose (MID) test, 20 Kunming mice weighing 18–22 g were used (10 males and 10 females). The animals were fasted for 16 h before the test and were not allowed to access drinking water. Corn oil was used as a solvent because of the poor solubility of astaxanthin in water. The astaxanthin sample in the amount of 20.0 g was added to 40 mL of corn oil and mixed well. This 500 mg/mL suspension was administered to animals through gavage twice with 6 h interval at 0.4 mL/20 g BW each time. The total dose was 20 g/kg BW. After the gavage, the animal’s poisoning symptoms were observed and recorded. The animals were observed for two weeks and weighed once a week. At the end of the experiment, the animals were euthanized and dissected for general observation. The acute toxicity of the test reagent was evaluated according to the toxicity grading standards.

### 2.3. Ames Test

The identified strains of histidine-deficient *Salmonella typhimurium* strains TA97a, TA98, TAl00 and TA102 were used in the test. The sample in the amount of 5.0 g was added to 100 mL of dimethyl sulfoxide and mixed to make the 50 mg/mL stock solution. This stock solution was then serially diluted five times each (10 mL plus dimethyl sulfoxide to 50 mL) to make the 10, 2, 0.4, 0.08 mg/mL solutions, which were autoclaved (0.103 MPa, 20 min) for sterilization and used for testing. Rat liver microsomal shade (S9) induced by polychlorinated biphenyls was used as an in vitro metabolic activation system. The plate incorporation method was used. To make the incubated top culture medium, 0.l mL of the test strain enrichment solution, 0.1 mL of the test substance solution and 0.5 mL of the S9 mixture solution (metabolic activation system) were added and mixed well. This mixture was poured onto the bottom medium plate. The five test doses were 5000, 1000, 200, 40 and 8 μg/dish (DG). In addition, spontaneous (NG), solvent (SG), and positive mutant controls (PG) were also set. The spontaneous reversal control was the same as the sample group except that no sample was added. The solvent control was replaced with dimethyl sulfoxide, and the rest of the conditions were the same as those of the sample group. Each strain of each dose group was performed in triplicate. The culture was carried out at 37 °C for 48 h, and the number of colonies per dish was counted. The entire set of tests was repeated twice under the same conditions. If the number of revertant colonies of the test substance increases to more than twice the number of spontaneously reverted colonies and has a dose–response relationship, the mutagenesis test is positive.

### 2.4. Micronucleus Test of Polychromatic Erythrocytes (PCE)

Fifty Kunming mice with a body weight of 25–30 g were randomly divided into five groups, 10 in each group (five males and five females). The three doses of the test group were set at 10,000, 5000, 2500 mg/kg BW (A-1–A-3). Corn oil was used as the negative control, and a 40 mg/kg BW dose of cyclophosphamide (cp) was used as the positive control. Samples in the amount of 20.0, 10.0 and 5.0 g were added to 40 mL of corn oil and mixed well to make the 500, 250, 125 mg/mL suspensions. They were administered to the animals via gavage at 0.4 mL/20 g BW. The negative control group (NC) was given an equal volume of corn oil and the positive control group (PC) was given an equal volume of the 2 mg/mL cyclophosphamide solution.

### 2.5. Mouse Sperm Abnormality Test

Fifty Kunming male mice weighing 25–35 g were randomly divided into five groups (10 in each group), test groups for three doses (10,000, 5000, 2500 mg/kg BW,) the negative control group (corn oil) and the positive control group (40 mg/kg BW dose of cyclophosphamide (cp)). Astaxanthin, the negative and positive control solutions used here were prepared as indicated in the previous section (Section 2.4). They were administered to the animals via gavage at 0.4 mL/20 g BW and once a day for five consecutive days. The animals were sacrificed on the 30th day after the last dose, and the sperm of the epididymis was smeared, fixed in methanol and stained with eosin. Under an optical microscope, 1000 sperm counts per animal were counted to determine the deformity, and the sperm deformity rate was calculated.

### 2.6. Lymphocyte Transformation Test

The MTT (3-(4,5-dimethylthiazol-2-yl)-2,5-diphenyltetrazolium bromide) assay was used in the ConA-induced mouse spleen lymphocyte transformation test. The spleen was taken aseptically, placed in a dish with a suitable amount of sterile Hanks’ solution and gently grounded with tweezers to prepare a single-cell suspension. The resulting mixture was filtered through a 200 mesh cell strainer and washed twice with Hanks’ solution after centrifugation at 1000 rpm for 10 min. The cells were then resuspended in 1 mL of the complete medium, and the number of viable cells (should be above 95%) was counted by phenol blue staining to adjust the cell concentration to 3 × 10^6^ cells/mL. Each spleen cell suspension was added to a 24-well culture plate in two wells, 1 mL per well, one well plus 75 μL of the ConA solution (equivalent to 7.5 μg/mL), and the other well was used as the control. They were incubated at 37 °C at 5% CO_2_ for 72 h in a CO_2_ incubator. For the MTT assay, 0.7 mL of the culture medium per well was gently aspirated, then 0.7 mL of the RPMI 1640 medium containing no calf serum was added, and 50 μL/well of MTT (5 mg/mL) was added to each well, and the cells were incubated for 4 h. After the completion of the culture, 1 mL of acidic isopropanol was added to each well to terminate and lyse the cells. The mixture was pipetted well to completely dissolve the purple crystals. Then, 100 μL/well of each mixture was added to 96-well plates in triplicate, and the optical density value was measured with a microplate reader at the wavelength of 570 nm to determine lymphocyte proliferation capacity.

### 2.7. Dinitrofluorobenzene (DNFB) Induces Delayed Type Hypersensitivity (DTH)

Ear swelling method: After sensitizing the mice with 1% DNFB (prepared with 1:1 acetone sesame oil solution), the right ear was treated with DNFB on the 5th day. After 24 h, the animals were sacrificed. A piece of tissue with the diameter of 8 mm from the left and right ears was obtained using an ear puncher. The tissue pieces were weighed, and the difference in weight between the left and right ears was used to present the degree of DTH.

### 2.8. Antibody-Producing Cell Count Test

Defibrinated sheep blood was washed three times with saline and centrifuged at 2000 rpm for 10 min each time. Each mouse received an intraperitoneal injection of 0.2 mL 2% (*v*/*v*) SRBC (sheep red blood cells). The mice were sacrificed 4–5 days after SRBC immunization, and the spleen was removed and placed in a small dish containing Hanks’ solution. The spleen was gently grounded to prepare a suspension of cells, which was filtered through a 200 mesh cell strainer and centrifuged at 1000 rpm for 10 min. The cell pellet was washed twice with Hanks’ solution and eventually suspended in 5 mL of the RPMI 1640 medium. Cell numbers were counted, and the final cell concentration was adjusted to 5 × 10^6^ cells/mL.

Determination of plaque formation: To make the surface medium, 1 g of agarose was added to 100 mL of double-distilled water and heated to dissolve agarose. The mixture was placed in a 45–50 °C water bath and mixed with an equal volume of the two-times concentrated Hanks’ solution at pH 7.2~7.4, which was aliquoted into small tubes at 0.5 mL per tube. After that, 50 μL of 10% SRBC (*v*/*v*, prepared with the SA buffer) and 20 μL of the spleen cell suspension (5 × 10^6^ cells/mL) were added, mixed rapidly and poured onto a thin layer of agarose on glass slides. Parallel slices were made. After the agar solidified, the slides were placed horizontally on a rack, incubated in a carbon dioxide incubator for 1.5 h. Then, the diluted complement in the SA buffer (1:8) was added to the groove of the slide holder. The slides were incubated for another 1.5 h. The number of hemolysis plaques was counted.

### 2.9. Determination of Serum Hemolysin

Sheep blood was taken and washed three times with saline after it was centrifuged at 2000 rpm for 10 min each time. The SRBC pellet was resuspended into the 2% (*v*/*v*) cell suspension in saline. Each mouse received an intraperitoneal injection of 0.2 mL of this suspension for immunization. After 4–5 days, the mouse eyeballs were removed for blood collection. The collected blood was stored in a centrifuge tube and allowed to stand for one hour. The coagulated blood was peeled off from the tube wall, and the serum was collected after centrifugation at 2000 rpm for 10 min.

Agglutination reaction: The serum was diluted in saline, and the serum of different dilutions was placed in a hemagglutination test microplate, 100 μL per well. Then, 100 μL of the 0.5% (*v*/*v*) SRBC suspension was added, mixed and placed onto the wet flat plate with a cover. Then, the plates were incubated at 37 °C for 3 h. The degree of blood cell agglutination was recorded. The antibody volume number was calculated based on the level of serum coagulation.

### 2.10. Mouse Carbon Clearance Test

Diluted Indian ink (10 mL/kg) was injected to the tail vein of the mouse according to body weight. After the ink injection, a sample of 20 μL of blood was taken from the internal iliac venous plexus at 2 or 10 min and immediately added to 2 mL of a 0.1% Na_2_CO_3_ solution. From each sample, 0.1 mL were taken and put into a 96-well microtiter plate, and the optical density value (OD) was measured at the wavelength of 600 nm using a microplate reader. The empty Na_2_CO_3_ solution was used as the negative control.

After that, those mice were sacrificed for the collection of the liver and spleen samples. The mass of these organs was weighed after removal of absorption of blood using filter paper.

The ability of a mouse to clear the carbon profile is determined by the phagocytic index. The phagocytic index is calculated as follows. When the phagocytic index of the test sample group was significantly higher than that of the control group, the experimental result was considered to be positive.

Phagocytic index = final weight × K^1/3^/(liver weight + spleen weight) (1)

K = (logOD_1_ − logOD_2_)/(t_2_ − t_1_) (2)

### 2.11. Peritoneal Macrophage Phagocytosis of the Chicken Red Blood Cells Test

Semi-in vivo method: The 20% chicken red blood cells suspension was prepared as described [18]. Each mouse was intraperitoneally injected with 1 mL of this suspension. After 30 min, the animals were sacrificed and set on a plate for laparotomy. After exposure of the abdominal cavity, 2 mL of normal saline was injected intraperitoneally, and the plate with the mouse was swirled for 1 min. After that, 1 mL of a peritoneal washing solution was taken and dispensed evenly on two slides, which were incubated at 37 °C for 30 min. Then, the slides were rinsed with saline, air-dried, fixed in 1:1 acetone/methanol solution, stained in 4% Giemsa–phosphate buffer dye for 3 min, rinsed again with distilled water and dried. Under the microscopic oil lens, 100 macrophages were counted and used to calculate the phagocytic rate and phagocytic index as described [19].

### 2.12. NK Cell Activity

Subculture of target cells (YAC-1 cells): The target cells were subcultured for 24 h before the experiment. The cells were washed three times with Hanks’ solution and adjusted to the cell concentration of 4 × 10^5^ cells/mL with the RPMI 1640 complete medium.

Preparation of the spleen cell suspension (effector cells): The spleen was aseptically placed in a small dish containing an appropriate amount of sterile Hanks’ solution, and the spleen was gently crushed with tweezers to prepare a single-cell suspension. The suspension was filtered through a 200 mesh cell strainer and washed twice with Hanks’ solution at 1000 rpm for 10 min. The supernatant was removed and the pellet was tapped to suspend. After that, 0.5 mL of sterilized water were added to lyse red blood cells for 20 s, which was followed by additions of 0.5 mL of two-times Hanks’ solution and 8 mL of Hanks’ solution. This mixture was spun at 1000 rpm for 10 min, and the pellet was resuspended in 1 mL of RPMI l640 containing 10% calf serum. The culture medium suspension was diluted with 1% glacial acetic acid, and the final cell concentration was adjusted with the RPMI l640 complete medium to reach 2 × 10^7^ cells/mL. The number of viable cells was counted by phenol blue staining (should be above 95%).

NK cell activity assay: For the effector/target (50:1 ratio) group, 100 μL of the target cells and 100 μL of effector cells were added into a well of a U-shaped 96-well culture plate. For the basal release group, 100 μL of the target cells and 100 μL of the culture medium were mixed in one well. For the maximum release group, 100 μL of the target cells and 100 μL of 1% NP40 were mixed in one well. All the three groups were performed in triplicate and incubated at 37 °C and 5% CO_2_ for 4 h. Then, the 96-well culture plate was centrifuged at 1500 rpm for 5 min. From each well, 100 μL of the supernatant were transferred to a well of a flat bottom 96-well culture plate, and then 100 μL of an LDH substrate solution were added. The reaction in the mixture was carried out for 3–10 min at room temperature before 30 μL of 1 mol/L HCl was added to each well. The optical density value (OD) was measured at 490 nm.

The NK cell activity was calculated as described. When the NK cell activity of the test sample group was significantly higher than that of the control group, the result of the experiment was considered to be positive [20].

### 2.13. Statistical Analysis

Statistics were performed using the SPSS 11.5 software. All the data were analyzed using ANOVA. When the variance was homogeneous, one-way ANOVA was used. When the variance was uneven, the rank-sum test was used.

## 3. Result and Discussion

### 3.1. Acute Toxicity

After administration of a dose of 20 g/kg BW, the animals grew well, and astaxanthin treatment did not affect the gain of body weight (Table 1). No symptoms of poisoning were observed in the mice tested, and no animal deaths were observed for 14 days. At the end of the experiment, the animals were euthanized and dissected. Gross inspection did not identify obvious abnormal changes in the main organs such as the liver, kidneys, spleen, heart, lungs, stomach and intestines. The results showed that the maximum tolerated dose (MTD) of the sample’s acute oral toxicity was greater than 20 g/kg BW in mice, and the acute oral toxicity was non-toxic.

### 3.2. Ames Test

Table 2 shows that for the four test strains TA97a, TA98, TA100 and TA102, regardless of the presence or absence of astaxanthin, the number of retrograde colonies in each dose group was not higher than twice the number of spontaneous retrograde colonies, showing no dose–response relationship. The results show that the mutagenesis test of the tested strain was negative. 

### 3.3. Micronucleus Test of Polychromatic Erythrocytes (PCE) in the Mice

**Figure 1:**
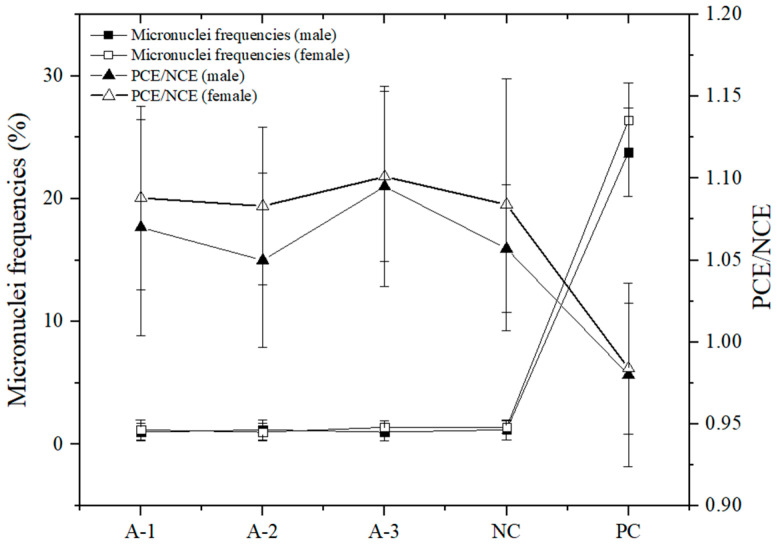
Effect of astaxanthin on the incidence of micronuclei in bone marrow cells of mice. A-1: 10,000 mg/kg BW; A-2: 5000 mg/kg BW; A-3: 2500 mg/kg BW; NC: negative control group; PC: positive control group.

Figure 1 shows that there were no significant differences in the micronucleus rate of bone marrow cells between each dose group and the negative control group (*p* > 0.05). The PCE/NCE value of each dose group was not less than 20% of that of the negative control group, and there were no significant differences compared to the negative control group. The micronucleus rate of the cyclophosphamide positive control group was not significantly different from that of the negative control group. The difference between the positive and negative control groups was dramatically significant (*p* < 0.01), suggesting that the samples had no damage or inhibition of bone marrow cells in mice.

### 3.4. Sperm Abnormality Assay

**Figure 2:**
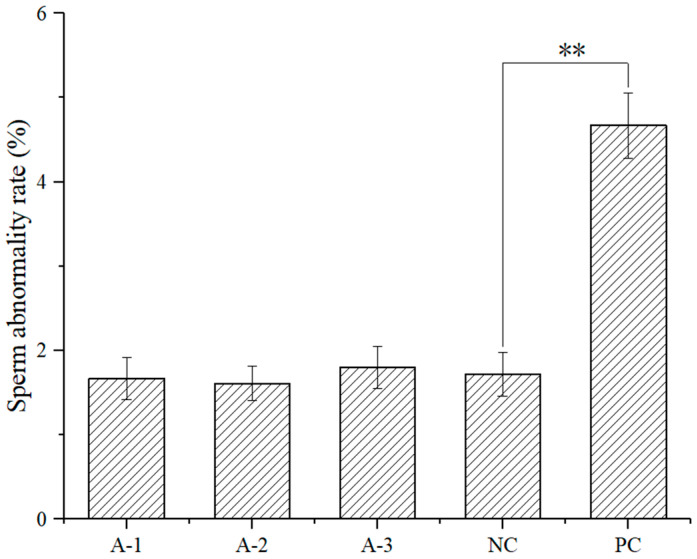
Effect of astaxanthin on the sperm abnormality rate. ** *p* < 0.01.

As shown in Figure 2, there were no significant changes in the incidence of sperm abnormality in mice. There were no significant differences in the sperm abnormality rate between each dose group and the negative control group (*p* > 0.05). However, there was a significant difference between the cyclophosphamide positive control group and the negative control group (*p* < 0.01), suggesting that no sperm abnormality was observed in the male mice treated with astaxanthin.

### 3.5. Effect of Astaxanthin on the Immune Organ/Body Weight Ratio of the Mice

**Figure 3:**
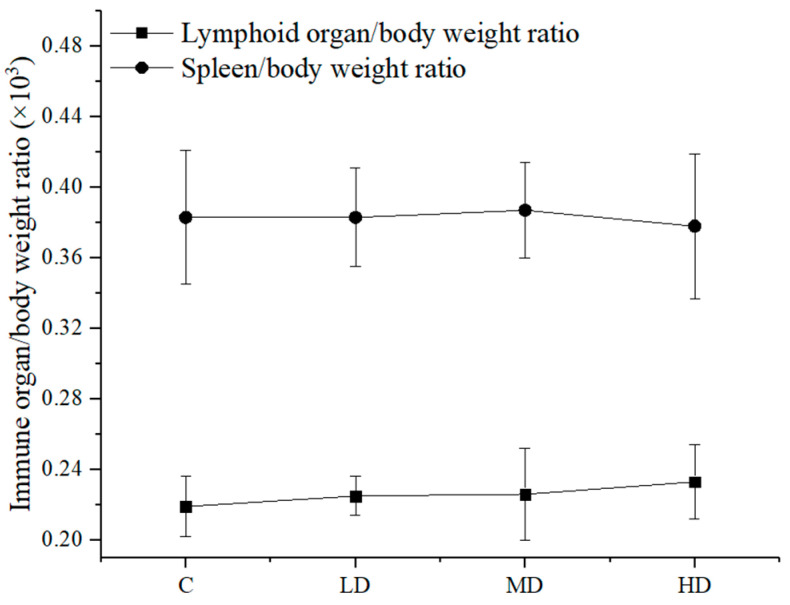
Effect of astaxanthin on the incidence of sperm abnormality in the mice. C: control group. LD: low dose group (84 mg/kg BW). MG: medium dose group (167 mg/kg BW). HD: high dose group (334 mg/kg BW).

Different doses of astaxanthin from *Haematococcus pluvialis* were orally administered to mice to determine the ratio of immune organs to body weight in the mice. The results are shown in Figure 3.

Figure 3 shows that there was no significant difference in the thymus/body weight ratio and the spleen/body weight ratio between the groups (*p* > 0.05).

### 3.6. Effect of Astaxanthin on the Cellular Immune Function in the Mice

**Figure 4:**
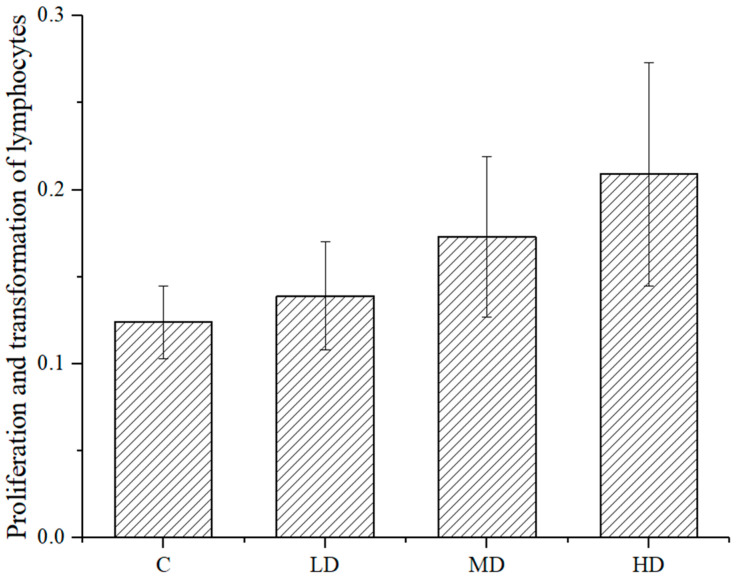
Results of astaxanthin transformation in mouse spleen lymphocytes.

As shown in Figure 4, the proliferation and transformation ability of mouse spleen lymphocytes in each dose group was higher than that in the negative control group, and the difference between the high- or medium-dose groups and the negative control group was significant (*p <* 0.01 and *p* < 0.05, respectively). This astaxanthin sample promoted the proliferation and transformation ability of spleen lymphocytes of the mice.

**Figure 5:**
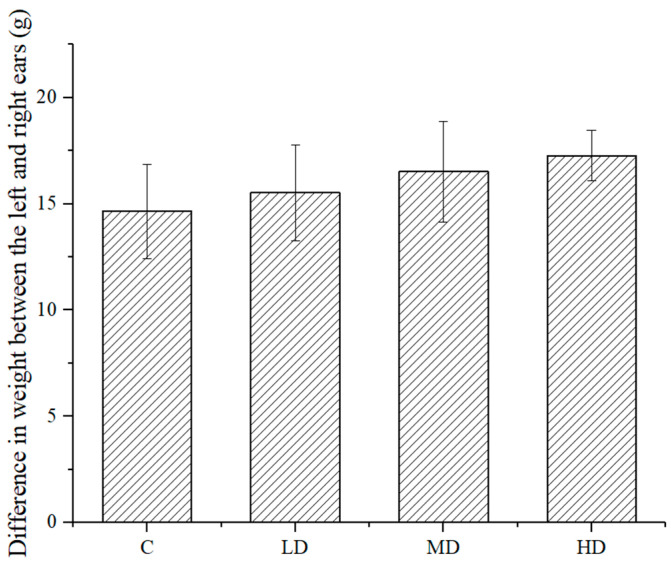
Experimental results of astaxanthin in the mice with a delayed allergic reaction.

As shown in Figure 5, the difference in weight between the left and right ears of the mice in each dose group was higher than that in the negative control group. The difference between the high-dose group and the negative control group was significant (*p* < 0.05), indicating that the sample treatment promoted delayed type hypersensitivity.

### 3.7. Effect of Astaxanthin on Serum Hemolysin in the Mice

**Figure 6:**
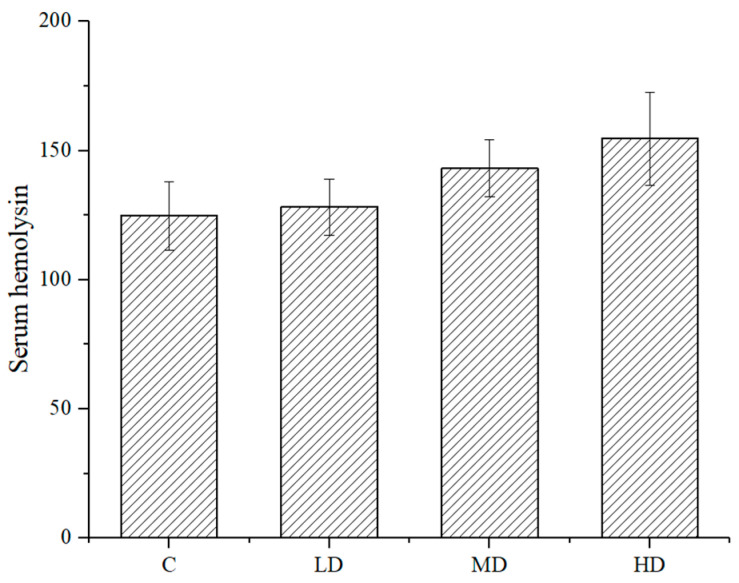
Effect of astaxanthin on serum hemolysin in mice.

As shown in Figure 6, the antibody volume of the mice in each dose group of astaxanthin was higher than that of the negative control group. The difference between the high- or medium-dose group and the haze control group was significant (*p* < 0.01 and *p* < 0.05, respectively), indicating that the astaxanthin treatment did not increase the serum hemolysin level in the mice.

### 3.8. Effects of Astaxanthin on Phagocytosis of Peritoneal Macrophages

As shown in Table 3, the phagocytic rate and phagocytic index of mouse erythrocytes in the astaxanthin-treated mice were higher than those in the negative control group. There was no significant difference between the dose groups and the negative control group (*p* > 0.05), indicating that astaxanthin had no significant effect on the phagocytic function of mouse peritoneal macrophages.

### 3.9. Effect of Astaxanthin on Carbon Particle Clearance in the Mice

Table 4 shows that, compared with the blank control group, the carbon particle clearance function of the mice in each dose group of astaxanthin was higher than that of the negative control group. The difference between the high- or medium-dose group and the sputum control group was significant (*p* < 0.01 and *p* < 0.05), indicating that astaxanthin can significantly increase carbon particle clearance in mice.

### 3.10. Effect of Astaxanthin on the Function of Antibody-Producing Cells

As shown in Table 5, there were more antibody-producing cells in the astaxanthin-treated mice than in the negative control group. The difference between the high- or medium-dose group and the sputum control group was significant (*p* < 0.01 and *p* < 0.05, respectively), indicating that astaxanthin can significantly enhance the ability of antibody-producing cells.

### 3.11. Effect of Astaxanthin on the Activity of Mouse NK Cells

As shown in Table 6, the activity of NK cells in each dose group of astaxanthin was higher than that in the control group. The high-dose group could significantly enhance the activity of NK cells compared with the blank control group in mice (*p* < 0.05).

Here, the experiments were carried out to determine the effects of astaxanthin derived from *Haematococcus pluvialis* on immune functions in mice, which covered three areas: cellular immunity, humoral immunity and nonspecific immunity. According to the recommended 0.02 g/kg BW intake of erythrophytic astaxanthin for humans, the experimental doses were increased 10, 20 and 30 times to set the low-, medium- and high-dose groups, respectively. The experimental results show that a high dose can significantly enhance the ConA-induced rat spleen lymphocyte proliferation ability, delayed dysfunction of mice induced by dinitrofluorobenzene, serum hemolysin content in mice and activities of antibody-producing cells. The results of four tests show that astaxanthin can enhance cellular and humoral immune functions. In terms of nonspecific immunity, the experimental results show that astaxanthin can promote the mononuclear macrophage carbon particle clearance rates in mice and mouse peritoneal macrophages-mediated phagocytosis of chicken red blood cells, which can increase NK cell activity at a high dose. Nonspecific immunity is also enhanced dramatically. These results are basically consistent with the conclusions made by others.

Some researchers believe that astaxanthin can improve the differentiation and proliferation of B lymphocytes in sports injury model animals and significantly increase the levels of serum immunoglobulins IgA, IgM and IgG (*p* < 0.01), showing the benefits on humoral immunity. Therefore, we studied the immunopotentiation effect of astaxanthin in mice [21].

Sergio et al. [22] extracted astaxanthin from shrimps and treated *Helicobacter pylori*-infected mice with it. The results showed that the treatment with this astaxanthin had increased secretion of interferon gamma, interleukin 1 and interleukin 2 from spleen leukocytes of the infected mice. They demonstrated that astaxanthin extracted from shrimps has the potential to increase humoral immunity.

Some scholars have fed astaxanthin to aquatic products such as sea prawns and found that after feeding, the resistance of sea prawns to pathogenic microorganisms is enhanced, which is attributed to the mechanism of enhanced humoral immunity [23]. Astaxanthin can partially restore functions of the humoral immune system in aged mice and increase the IgM, IgA and IgG levels, indicating that astaxanthin treatment enhances humoral immunity at the early stage of antigen invasion [24]. Others have reported that astaxanthin can stimulate the proliferation of T lymphocytes in immunocompromised model animals, showing that astaxanthin functions to promote cellular immunity [25].

At present, the research on the immunopotentiation effect of astaxanthin is limited and usually only involves a certain aspect of humoral immunity, cellular immunity and specific immunity. No systematic and complete research articles were found. In this article, the effects of astaxanthin from *Haematococcus pluvialis* on cellular immunity, humoral immunity and nonspecific immunity were studied thoroughly and systematically. We concluded that the treatment with astaxanthin from *Haematococcus pluvialis* promotes cellular immunity, humoral immunity and nonspecific immunity in mice.

Astaxanthin is often used in healthy human populations. However, the majority of the published research papers use model animals, but not normal animals, as experimental subjects, which is not in line with the practical application of astaxanthin. In this article, animals in the normal physiological state were used, and the effects of astaxanthin on the immune system are more representative of normal conditions.

The mechanism by which astaxanthin enhances immunity has not been reported. It is speculated that it may be closely related to the antioxidant activities of astaxanthin.

Singlet oxygen has a cytotoxic effect on the animal’s immune system. It facilitates the production of free radicals that lead to the degradation of macrophage cell membranes, resulting in decreased phagocytic efficiency and antigen presentation capability [26]. Free radicals can also attack T lymphocytes and B lymphocytes. T lymphocytes mediate cellular immunity, and their activation and proliferation are central to the immune response [27]. After being stimulated by an antigen, T lymphocytes stimulate an immune response and clear the antigen. After B cells are stimulated, they can produce antibodies such as IgG, IgA, IgE and IgM, which bind to antigens and clear them [28]. When these two immune cells are attacked by free radicals, the entire immune system is affected. Astaxanthin can quench singlet oxygen, directly scavenge oxygen-free radicals, stabilize the immune cell membrane structure and enhance immunity by protecting the integrity of various immune cells [29].

*Haematococcus pluvialis* is a kind of edible algae. The toxicity research of astaxanthin derived from *Haematococcus pluvialis* has not received enough attention. Only a few research groups have conducted toxicological studies of astaxanthin derived from *Haematococcus pluvialis*. Chintong [30] used cells to study astaxanthin toxicology and added the astaxanthin derived from shrimp shells to human skin fibroblasts. The cell morphology and quantity did not change. It shows the lack of toxic effect of the shrimp-derived astaxanthin on human skin fibroblasts. However, the most ideal way for toxicological experiments is to use whole animals as experimental subjects and study the effect at the cellular level. The toxicity of the tested drugs may be masked by the animal self-protection system. Therefore, some groups have studied the genotoxicity of astaxanthin. Results of the *Salmonella typhimurium* reverse mutation test, the micronucleus test and the sperm abnormality test have demonstrated that astaxanthin treatment does not cause genotoxicity. These several toxicological experiments have demonstrated that astaxanthin is safe and edible to a certain extent even without complete toxicological studies. In this article, the acute toxicity and genotoxicity tests were conducted in the mice treated with astaxanthin from *Haematococcus pluvialis* to evaluate its safety thoroughly and systematically.

## 4. Conclusions

Astaxanthin is one of the most powerful antioxidants and has been widely and deeply studied for its antioxidation properties. However, there are few studies on the safety and immune function. In this paper, the immune function of astaxanthin, including humoral immunity, cellular immunity and nonspecific immunity, and its safety were studied. The results showed that it was non-toxic, non-teratogenic and featured no damage or inhibition of bone marrow cells in the mice. In terms of cellular immunity, astaxanthin can promote the proliferation and transformation ability of spleen lymphocytes of mice, and can also promote the delayed type hypersensitivity. In the aspect of humoral immunity, astaxanthin can increase the level of serum hemolysin and promote the production of antibodies. In terms of specific immunity, astaxanthin had no significant effect on the phagocytic function of peritoneal macrophages in the mice, but it could significantly increase the phagocytic index of carbon clearance and the activity of NK cells in the mice.

## Figures and Tables

**Table 1:** The results of the acute toxicity test of the mice.

Sex	Baseline Weight (g)	Weight on the 7th Day (g)	Weight on the 14th Day (g)	MTD (g/kg BW)
Male	20.5 ± 1.1	27.2 ± 1.1	33.4 ± 1.1	>20
Female	20.3 ± 1.0	26.0 ± 1.3	31.2 ± 1.5	>20

**Table 2:** Mutagenicity of astaxanthin in the Ames test (M ± SD).

Groups	Dose (μg/plate)	TA97a		TA98		TA100		TA102	
		+S9	−S9	+S9	−S9	+S9	−S9	+S9	−S9
DG	5000	144.7 ± 15.4	140.0 ± 12.1	39.3 ± 7.5	39.0 ± 7.9	166.7 ± 14.6	172.0 ± 16.4	276.0 ± 17.1	273.0 ± 27.5
	1000	138.7 ± 14.6	145.3 ±11.0	38.3 ± 4.7	41.7 ± 6.4	158.7 ± 16.9	161.0 ± 15.0	275.3 ± 21.5	267.7 ± 20.5
	200	134.7 ± 14.4	142.0 ± 12.3	41.7 ± 9.1	40.0 ± 8.5	158.0 ± 19.1	161.3 ± 10.0	274.7 ± 23.5	269.0 ± 22.6
	40	135.7 ± 12.2	144.7 ± 17.6	41.3 ± 6.5	40.3 ± 5.1	160.0 ± 17.8	167.3 ± 11.4	279.0 ± 17.6	275.0 ± 21.7
	8	144.7 ± 18.0	140.7 ± 17.2	42.0 ± 5.6	40.0 ± 9.5	166.7 ± 18.0	161.3 ± 15.5	275.0 ± 20.7	274.3 ± 20.4
NG		136.7 ± 15.5	137.3 ±13.0	39.3 ± 7.1	38.7 ± 8.3	160.0 ± 17.4	156.7 ± 18.0	272.0 ± 16.6	280.0 ± 20.3
SG		138.3 ±13.2	141.0 ± 15.7	40.7 ± 5.7	41.3 ± 7.4	168.0 ± 13.1	164.7 ± 19.7	269.0 ± 22.5	274.3 ± 20.9
PG		2800.3 ± 106.5	1794.0 ± 62.5	3025.0 ± 93.7	4944.3 ± 131.6	3022.0 ± 209.1	2981.0 ± 107.6	888.0 ± 35.8	906.0 ± 42.9

DG: 5000, 1000, 200, 40 and 8 μg/plate dose groups. NG: negative group without any treatment. SG: solvent group with deionized water. PG: positive control group containing 2-AF (10.0 μg/plate for TA97, TA98, TA100 with S9), 9-fluorenone (0.2 μg/plate for TA97a, TA98 without S9), sodium azide (1.5 μg/plate for TA100 without S9), 1,8-dihydroxyanthraquinone (50.0 μg/plate for TA102 with S9) or mitomycin C (MMC) (0.5 μg/plate for TA102 without S9). These test conditions of the PG were set and implemented in order to confirm the reversion properties of each tested strain, and the efficacy of the metabolic activation system was measured in the presence (+S9) or absence of metabolic activation (−S9).

**Table 3:** Effect of astaxanthin on phagocytosis of mouse peritoneal macrophages.

Dose (mg/kg BW)	Phagocytic Ratio (%)	*p*	Phagocytic Index	*p*
334	25.1 ± 2.6	0.172	0.52 ± 0.05	0.234
167	24.6 ± 2.6	0.328	0.51 ± 0.08	0.287
84	24.1 ± 3.2	0.568	0.50 ± 0.07	0.571
0.0	22.7 ± 3.2		0.46 ± 0.09	

**Table 4:** Effect of astaxanthin on carbon particle clearance in the mice.

Dose (mg/kg BW)	Phagocytic Index	*p*
334	9.41 ± 0.89	0.023
167	9.39 ± 1.07	0.047
84	8.74 ± 0.87	0.494
0.0	8.24 ± 0.93	

**Table 5:** Effect of astaxanthin on antibody-producing cell functions.

Dose (mg/kg BW)	Phagocytic Index	*p*
334	206.6 ± 28.2	0.020
167	196.5 ± 24.5	0.119
84	179.6 ± 29.9	0.822
0.0	170.7 ± 29.8	

**Table 6:** Effect of astaxanthin on the activity of mouse NK cells.

Dose (mg/kg BW)	Activity of NK Cells	*p*
334	23.18 ± 3.58	0.095
167	21.49 ± 7.06	0.375
84	19.94 ± 6.03	0.727
0.0	17.43 ± 4.57	

## Data Availability

The study did not report any data.

## References

[1] Kaha M., Iwamoto K., Yahya N.A., Suhaimi N., Sugiura N., Hara H., Othman N., Zakaria Z., Suzuki K. (2021). Enhancement of astaxanthin accumulation using black light in Coelastrum and *Monoraphidium* isolated from Malaysia. Sci. Rep..

[2] Yamashita E. (2021). Extensive Bioactivity of Astaxanthin from Haematococcus pluvialis in Human. Adv. Exp. Med. Biol..

[3] Dansou D.M., Wang H., Nugroho R.D., He W., Zhao Q., Zhang J. (2021). Assessment of Response to Moderate and High Dose Supplementation of Astaxanthin in Laying Hens. Animals.

[4] Lim K.C., Yusoff F.M., Shariff M., Kamarudin M.S. (2021). Dietary astaxanthin augments disease resistance of Asian seabass, Lates calcarifer (Bloch, 1790), against *Vibrio alginolyticus* infection. Fish Shellfish. Immunol..

[5] Jiang S., Tong S. (2019). Advances in astaxanthin biosynthesis in *Haematococcus pluvialis*. Sheng Wu Gong Cheng Xue Bao.

[6] Raza S.H.A., Naqvi S.R.Z., Abdelnour S.A., Schreurs N., Mohammedsaleh Z.M., Khan I., Shater A.F., Abd El-Hack M.E., Khafaga A.F., Quan G. (2021). Beneficial effects and health benefits of Astaxanthin molecules on animal production: A review. Res. Vet. Sci..

[7] Alesci A., Salvo A., Lauriano E.R., Gervasi T., Palombieri D., Bruno M., Pergolizzi S., Cicero N. (2015). Production and extraction of astaxanthin from *Phaffia rhodozyma* and its biological effect on alcohol-induced renal hypoxia in *Carassius auratus*. Nat. Prod. Res..

[8] Higuera-Ciapara I., Félix-Valenzuela L., Goycoolea F.M. (2006). Astaxanthin: A review of its chemistry and applications. Crit. Rev. Food Sci. Nutr..

[9] Novoveská L., Ross M.E., Stanley M.S., Pradelles R., Wasiolek V., Sassi J.F. (2019). Microalgal Carotenoids: A Review of Production, Current Markets, Regulations, and Future Direction. Mar. Drugs.

[10] Oslan S.N.H., Shoparwe N.F., Yusoff A.H., Rahim A.A., Chang C.S., Tan J.S., Oslan S.N., Arumugam K., Ariff A.B., Sulaiman A.Z. (2021). A Review on Haematococcus pluvialis Bioprocess Optimization of Green and Red Stage Culture Conditions for the Production of Natural Astaxanthin. Biomolecules.

[11] Pereira A.G., Otero P., Echave J., Carreira-Casais A., Chamorro F., Collazo N., Jaboui A., Lourenço-Lopes C., Simal-Gandara J., Prieto M.A. (2021). Xanthophylls from the Sea: Algae as Source of Bioactive Carotenoids. Mar. Drugs.

[12] Lu Q., Li H., Zou Y., Liu H., Yang L. (2021). Astaxanthin as a microalgal metabolite for aquaculture: A review on the synthetic mechanisms, production techniques, and practical application. Algal Res..

[13] Fakhri S., Abbaszadeh F., Dargahi L., Jorjani M. (2018). Astaxanthin: A mechanistic review on its biological activities and health benefits. Pharmacol. Res..

[14] Ambati R.R., Phang S.M., Ravi S., Aswathanarayana R.G. (2014). Astaxanthin: Sources, extraction, stability, biological activities and its commercial applications—A review. Mar. Drugs.

[15] Pereira C.P.M., Souza A.C.R., Vasconcelos A.R., Prado P.S., Name J.J. (2021). Antioxidant and anti-inflammatory mechanisms of action of astaxanthin in cardiovascular diseases (Review). Int. J. Mol. Med..

[16] Shin J., Saini R.K., Oh J.W. (2020). Low Dose Astaxanthin Treatments Trigger the Hormesis of Human Astroglioma Cells by Up-Regulating the Cyclin-Dependent Kinase and Down-Regulated the Tumor Suppressor Protein P53. Biomedicines.

[17] Ni X., Yu H., Wang S., Zhang C., Shen S. (2017). Astaxanthin Inhibits PC-3 Xenograft Prostate Tumor Growth in Nude Mice. Mar. Drugs.

[18] Wu H., Niu H., Shao A., Wu C., Dixon B.J., Zhang J., Yang S., Wang Y. (2015). Astaxanthin as a Potential Neuroprotective Agent for Neurological Diseases. Mar. Drugs.

[19] Teselkin Y.O., Khoreva M.V., Veselova A.V., Babenkova I.V., Osipov A.N., Gankovskaya L.V., Vladimirov Y.A. (2018). Combined Effect of TLR2 Ligands on ROS Production by Mouse Peritoneal Macrophages. Bull. Exp. Biol. Med..

[20] Wang P.Y., Zhu X.L., Lin Z.B. (2012). Antitumor and Immunomodulatory Effects of Polysaccharides from Broken-Spore of *Ganoderma lucidum*. Front. Pharmacol..

[21] Jyonouchi H., Sun S., Gross M. (1995). Effect of carotenoids on in vitro immunoglobulin production by human peripheral blood mononuclear cells: Astaxanthin, a carotenoid without vitamin A activity, enhances in vitro immunoglobulin production in response to a T-dependent stimulant and antigen. Nutr. Cancer.

[22] Davinelli S., Melvang H.M., Andersen L.P., Scapagnini G., Nielsen M.E. (2019). Astaxanthin from Shrimp Cephalothorax Stimulates the Immune Response by Enhancing IFN-γ, IL-10, and IL-2 Secretion in Splenocytes of Helicobacter Pylori-Infected Mice. Mar. Drugs.

[23] Yu Y., Liu Y., Yin P., Zhou W., Tian L., Liu Y., Xu D., Niu J. (2020). Astaxanthin Attenuates Fish Oil-Related Hepatotoxicity and Oxidative Insult in Juvenile Pacific White Shrimp (*Litopenaeus vannamei*). Mar. Drugs.

[24] Jyonouchi H., Zhang L., Gross M., Tomita Y. (1994). Immunomodulating actions of carotenoids: Enhancement of in vivo and in vitro antibody production to T-dependent antigens. Nutr. Cancer.

[25] Jyonouchi H., Sun S., Iijima K., Gross M.D. (2000). Antitumor activity of astaxanthin and its mode of action. Nutr. Cancer.

[26] Muñoz M., Cedeño R., Rodríguez J., van der Knaap W.P.W., Mialhe E., Bachère E. (2000). Measurement of reactive oxygen intermediate production in haemocytes of the penaeid shrimp, *Penaeus vannamei*. Aquaculture.

[27] Valko M., Leibfritz D., Moncol J., Cronin M.T., Mazur M., Telser J. (2007). Free radicals and antioxidants in normal physiological functions and human disease. Int. J. Biochem. Cell Biol..

[28] Kamath B.S., Srikanta B.M., Dharmesh S.M., Sarada R., Ravishankar G.A. (2008). Ulcer preventive and antioxidative properties of astaxanthin from *Haematococcus pluvialis*. Eur. J. Pharmacol..

[29] Najafi H., Changizi Ashtiyani S., Sayedzadeh S.A., Mohamadi Yarijani Z., Fakhri S. (2015). Therapeutic effects of curcumin on the functional disturbances and oxidative stress induced by renal ischemia/reperfusion in rats. Avicenna J. Phytomed..

[30] Chintong S., Phatvej W., Rerk-Am U., Waiprib Y., Klaypradit W. (2019). In Vitro Antioxidant, Antityrosinase, and Cytotoxic Activities of Astaxanthin from Shrimp Waste. Antioxidants.

